# Do Migration Characteristics Influence the Utilization of Basic Public Health Services in Internal Elderly Migrants in China?

**DOI:** 10.3389/fpubh.2021.514687

**Published:** 2021-08-06

**Authors:** Yanwei Lin, Tingxian Wang, Tingting Zhu

**Affiliations:** ^1^Department of Health Sociology, School of Humanities and Management, Guangdong Medical University, Dongguan, China; ^2^School of Public Health, Guangdong Medical University, Dongguan, China

**Keywords:** internal elderly migrants, China, migration characteristics, social contacts, the utilization of basic public health services

## Abstract

**Background:** The literature shows that migration characteristics are a potential pathway through which migration can influence basic healthcare service utilization. The goal of the study was to explore the effect of migration characteristics on the utilization of basic public health services for internal elderly migrants in China and to identify the pathways that might promote their utilization of basic public health services.

**Methods:** We studied 1,544 internal elderly migrants. The utilization of basic public health services was determined through participation in free health checkups organized by community health service institutions in the past year. Migration characteristics were represented by years of residence and reasons for migration. Other variables included demographic characteristics and social factors, e.g., the number of local friends and exercise time per day were measured to represent social contacts. Multivariate binary logistic regression was employed to explore the association of the variables with the likelihood of using community health services.

**Results:** A total of 55.6% of respondents were men, and the mean age was 66.34 years (SD 5.94). A low level of education was observed. A total of 59.9% of migrants had been residents for over 10 years, and the main reason for migrating was related to family. Of these migrants, 12.9% had no local friends. Furthermore, 5.2% did not exercise every day. Social contacts were complete mediators of the impact of migration characteristics on the utilization of primary healthcare.

**Conclusion:** Our study highlighted the mediating role of social factors in the relationship between migration characteristics and the utilization of basic public health services among Chinese internal elderly migrants. The findings supported the need to increase the opportunities for social contacts between local elderly individuals and internal elderly migrants.

## Background

The utilization of basic public health services is an important aspect of the access migrants have to healthcare in the form of screening, preventive services, general practitioners, specialists, emergency rooms, and hospitals (1). In China, basic public health services are mainly provided by community health service centers (stations) in the community, with a large number of medical needs coming from large aging populations. One of the proposed solutions was to establish the elderly support systems in community health service centers (stations) through primary healthcare (2). The core functions of basic public health services in China that included prevention, case detection and management, gatekeeping, referral, care coordination, and so on were to be provided by community health service centers in urban communities (3, 4). This was expected to be an effective way to relieve the congestion of superior hospitals; however, unlike the hierarchical diagnosis and treatment system in developed countries, patients could choose different types of hospitals at will in China (5). Studies in developed countries have shown that immigrants have lower rates of health insurance and use less healthcare than local populations (2, 6). Specific determinants of health service utilization by immigrants were also inconsistent (7). Studies conducted in the Americas indicated that associations have been found between the length of stay and healthcare utilization of immigrants, and these findings suggested that acculturation or assimilation strongly correlated with the length of stay in the host society, which could be an important determinant of health status (8). However, anthropological studies thought that cultural differences did not necessarily fade with time (9).

Along with the increase in urbanization and development of the economy, internal migrants who move between regions within the country have gradually become an integral part of migration in the context of national labor shortages in China (10). The number of internal migrants reached 241 million in 2018, of which 18 million were more than 60 years of age according to the dynamic monitoring survey for internal migrants. Furthermore, the elderly migrant population over 60 years old was growing each year (11). As is known, household registration policy, commonly known as “hukou,” which was classified by origin into urban or rural “hukou,” was implemented in 1958 by Chinese authorities (12). “Hukou” gave households access to social benefits in their registration area but limited access to those outside their registration area (12, 13). Only migrants who started working for the government or were highly educated could change their “hukou” registration (14). In contemporary society, an increasing number of families are migrating with their elderly members to look after children, find jobs, or access better healthcare services in China. In addition, most of these elderly members are more than 60 years old (15). Strengthening the utilization of primary healthcare facilities is considered an effective approach to providing affordable, equitable access to quality basic health care for all Chinese citizens by 2020, as was pledged by China (13, 16). As the number of internal elderly migrants increases, their use of basic public health services should also improve to contribute to health equity. For example, as one of the basic public health services, the national policy proposed free health checkups in community health service centers (stations) for the elderly aged 65 each year, where these internal elderly migrants are not subject to household registration restrictions (17). However, many studies have indicated that <40% of elderly migrants have participated in the free community health checkups in the past year. In addition, <40% of internal elderly migrants follow up on chronic diseases, and the level of other behaviors, such as establishing health records and seeking medical attention, is also low (15, 18). This suggests that there are deficiencies in the health management of elderly migrants in China. In addition, this forms an institutional challenge for basic public health service utilization among elderly migrants. Therefore, it is necessary to explore how to improve the utilization of basic public health services among internal elderly migrants.

Studies have shown that the factors affecting the utilization of basic public health services for the elderly include demographic characteristics and social factors (2, 19, 20). Some studies believe that the migration characteristics of elderly migrants directly affect the utilization of health services (21), but other studies have suggested that migration characteristics are associated with basic public health service utilization through social adaption, acculturation, and other social factors (22). For example, migrating characteristics such as years of residence are one factor of the social support network and acculturation for migrants that contribute to basic public health service utilization (23). In general, it is evident that migrating characteristics and social factors, such as community engagement, social mobilization, ability to communicate, reason for migration, and length of stay in host countries, were related to health service delivery (24, 25). However, little work has been done to explore the possible pathways between social factors, migration characteristics, and the utilization of basic public health services, especially for internal elderly people (26–29). Therefore, our study explored the potential pathway of the impact of migration characteristics on the utilization of basic public health services for internal elderly migrants in China.

In our study, migration characteristics were represented by two dimensions: years of residence and reasons for migration, which were consistent with the literature (25). Social factors were represented by social contact, which plays an important role in determining individual health behaviors as a key dimension of poverty and well-being. However, the challenge of measuring social contact was daunting. A unified definition and measurement were not given by the vast and diverse conceptual literature on social contact (30). Empirical studies have explored different aspects of social contact, including physical isolation and access to social resources (31), such as “people feel that their communities” as a proxy for physical isolation and “ties with other people” as a proxy for access to social resources. However, these methods draw attention away from simply counting numbers of social contacts (32). For internal elderly people in China, due to retirement and migration, interactions with friends are the main social ties, and exercise in the community is the main way of feeling their communities. Referring to the literature and considering the actual situations of the population in question, the number of local friends and exercise time per day in the community were chosen as indicators of measuring two aspects of social contacts (33).

In this study, we presented the situation of the utilization of basic public health services for internal elderly migrants and explored the potential pathway through which migration characteristics impact the utilization of basic public health services. The objective of this study was to complement the existing literature by providing further insights into the pathway that might influence basic public health service utilization for internal elderly migrants. The results might help policymakers design appropriate social policies to promote the utilization of basic public health services in this disadvantaged population.

## Methods

### Data and Sampling

Data were derived from the dynamic monitoring survey for internal migrants—a special survey on internal elderly migrants of the National Health Commission of the People's Republic of China in 2015. The survey was organized by the National Health Commission in 2015 (formerly the National Health and Family Planning Commission). It was part of the regular data collection of the government, and face-to-face home-based interviews were conducted by the unified training investigators. The response rates for the survey were not announced by the governments. Our local institutional review board (IRB) exempted the analysis of the public-access data because it involved analyzing existing data that had been de-identified; ethical approval was not required for secondary data.

Stratified, multistage sampling based on a probability proportionate to size (PPS) sampling method was adopted. The basic sampling goal was all migrant households that did not have “hukou” (registered resident certificate) in the local area and had been living there for more than a month as reported by each village or neighborhood. Townships were randomly selected, followed by villages or neighborhoods. In each village or neighborhood, households of migrants were selected.

Eight pilot cities (Beijing, Shanghai, Dalian, Wuxi, Hangzhou, Hefei, Guangzhou, and Guiyang) were chosen as sampling cities. Regarding location: Beijing, Shanghai, Hangzhou, Guangzhou, and Wuxi are located in the east and are more economically developed, Guiyang belongs to the western region, Hefei is in the central region, and Dalian is in the northeast region. Finally, 16,960 migrant households, which included 1,544 (5.5%) internal elderly migrants aged over 60, participated in the survey. In view of the bias caused by different regions, regional classification was incorporated into the model as fixed effects to disaggregate the influence of different areas.

### Measures

Based on the existing data, considering that internal elderly migrants who moved between regions within the country and were more than 60 years old had reached the statutory retirement age, the informal networks of these migrants were their social ties. Since a unified definition and measurement of social contact was not given by literature, we chose the number of local friends and exercise time per day as the variables of social contacts following relevant researches (5, 7, 32). The respondents were asked how many friends they possessed in the local city; for comparative analysis, we adjusted the number of friends to a categorical variable with 7 categories: 0, 1–2, 3–4, 5–6, 7–8, 9–10, and 10 and above. The average daily exercise time was the time spent on physical exercise every day, such as walking more than 40 min, running, playing ball, aerobics, and swimming. If the respondents did not exercise every day, the weekly exercise time was divided by 7 to obtain the average daily exercise time. Similarly, the average daily exercise time was converted to 6 categories of categorical variables (0, within 30, 31–60, 61–90, 91–120 min, and over 120 min) for comparison in this study.

Furthermore, migration characteristics were represented by years of residence in the local city and reasons for migration (25). Demographic characteristics included age, gender, education, marital status, medical insurance, average monthly household income, and self-reported health (Table 1).

**Table 1:** Variable assignment.

**Variables**	**Assignment**
Gender	Male, Female
Age	60–64, 65–69, 70–74, 75–79, 80 and above
Education	Primary school or below, Middle and high schools, College and above
Marital status	Married, Single
Physical condition	Healthy, Basically healthy, Unhealthy, but can take care of themselves, Unhealthy, and cannot take care of themselves
Medical insurance	None, New Rural Cooperative Medical Scheme (NCMS), Urban and Rural Resident Cooperative Medical Insurance (URRCMI), Urban Resident Basic Medical Insurance (URBMI), Urban Employee Basic Medical Insurance (UEBMI), Free Medical Insurance (FMI)
Years of residence	1–6 months, 6 months−1 year, 1 year–, 2years–, 3years–, 4years–, 5years–, ≥10years
Reasons for migration	Working or engaging in trade, Taking care of children, Taking care of grandchildren, Therapy, Spending their remaining life with children, Other reason
Number of local friends	0, 1–2, 3–4, 5–6, 7–8, 9–10, 10 and above
Average exercise time per day	0, Within 30 min, 31–60 min, 61–90 min, 91–120 min, Over 120 min
Participate in free health checkup within the past year	No, Yes

### Utilization of Basic Public Health Services

Health management services, which include lifestyle and health status assessment, medical check-ups, auxiliary examinations, and health guidance, were clearly proposed in the basic public health services launched in 2009 in China (20). This project is considered to be of great significance in controlling chronic diseases of the elderly and improving the health of the elderly. The “National Basic Public Health Service Standards (2011 Edition)” retains and improves the above-mentioned content of the “Elderly Health Management Service Standards.” Furthermore, the document requires community health service centers (stations) to provide health management services for the elderly once a year as one of the basic public health services provided by community health service agencies (2). Free health checkups are still the core content of health management services for the elderly, and it is a prerequisite that the elderly enjoy follow-up basic public health services. Free health checkups include routine blood, blood pressure, blood lipid, fasting blood glucose, and blood uric acid tests, ECG examination, digital chest radiography (liver, ultrasonography of gallbladder and kidney, etc.), and screening for some malignant tumors. In recent years, the classification standard for elderly individuals who enjoy free health checkups is 60 years old and above in most areas, and they are not restricted by their place of household registration. In general, community health service centers (stations) usually provide free health checkup services once a year for elderly people over 60 years old in the community within a fixed period each year. The potential participants are informed by issuing leaflets, posting posters, calling through the telephone, and using other methods within the community. Recently, results showed that the percentage of migrant older adults receiving free medical checkups was 36.2% (15). Therefore, our study selected free health checkups as the measurement of basic public health services.

Whether elderly migrants participated in free health checkups within the past year provided by community health service agencies was adopted as an indicator of their utilization of basic public health services in the context of free medical check-up services provided to elderly people over 60 years old. If the respondent participated in free health checkups provided by community health service agencies within the past year, in other words, his answer was “Yes,” we thought basic public health services utilization occurred and vice versa.

### Statistical Analyses

Demographics, migrating characteristics, and social contacts were presented by descriptive analyses. We calculated the average and standard deviations in age, average monthly household income, and number of local friends. Furthermore, we conducted a classification process and calculated the frequency of each categorical variable. Chi-squared analysis was used to test the relevance of each categorical variable to the utilization of basic public health services. Multivariate binary logistic regression was developed to understand the associations of the variables with the likelihood of using basic public health services through modeling odds ratios. To explore the pathway of migrating characteristics and social contacts for basic public health services, three models were employed. First, the demographic variables were entered (model I), then the variables of migration characteristics were added (model II), and finally, the variables of social contacts were added (model III). In models II and III, we adjusted for demographic variables as fixed effects, and other independent variables were subjected to multifactor analysis using forward stepwise regression (forward: LR) based on the maximum likelihood estimation with a significance level of.05.

## Results

### Demographic Characteristics

A total of 1,544 internal elderly migrants were included across 4 districts. A total of 55.6% of internal elderly migrants were men, the mean age was 66.34 years (SD, 5.94), and 50.2% of the internal elderly migrants were aged 60–64 years old. On average, we observed a low level of education: 88.6% of the individuals had received high school education or less. A total of 78.2% were married, and 94.7% rated their health as healthy or basically healthy. Most of the respondents (74.7%) were in the eastern region. The 25–75% interquartile range of the average monthly household income of the migrants was 5,000–12,000 RMB (or US$784–1,881). More than half of them (52.5%) had New Rural Cooperative Medical Care Insurance (NCMS). This meant that the majority of internal elderly migrants came from rural areas, which was the result of the household registration policy (“hukou”) that people with rural household registration (rural “hukou”) can only participate in the NCMS in their hometown (“hukou” location). It was also found that 33% of internal elderly migrants had participated in a free medical checkup in community health service centers (stations) over the past year (Table 2).

**Table 2:** Demographic characteristics of respondents as internal elderly migrants (*n* = 1,544).

**Characteristics**	**Attending community free heath checkups**		**Total (%)**	**Frequency (Yes/No) (%)**
	**Yes (%)** ** (*n* = 511)**	**No (%) ** **(*n* = 1,033)**		
Gender^*^ (χ^2^ = 4.757, *p* = 0.029)
Male	304 (59.5)	554 (53.6)	858 (55.6)	35.4
Female	207 (40.5)	479 (46.4)	686 (44.4)	30.2
Age^**^ (χ^2^ = 13.28, *p* = 0.01)
60–64	229 (44.8)	546 (52.9)	775 (50.2)	29.6
65–69	132 (25.8)	255 (24.7)	387 (25.1)	34.1
70–74	75 (14.7)	133 (12.9)	208 (13.5)	36.1
75–79	54 (10.6)	71 (6.9)	125 (8.1)	43.2
80–	21 (4.1)	28 (2.7)	49 (3.2)	42.9
Education^***^ (χ^2^ = 18.124, *p* = 0.000)
Primary school or below	218 (42.7)	465 (45.0)	683 (44.2)	31.9
Middle and high schools	232 (45.4)	453 (43.9)	685 (44.4)	33.9
College and above	61 (11.9)	115 (11.1)	176 (11.4)	34.7
Marital status^***^ (χ^2^ = 1,544.000, *p* = 0.000)
Married	397 (77.7)	811 (78.5)	1,208 (78.2)	32.9
Single	114 (22.3)	222 (21.5)	336 (21.8)	33.9
Self-reported health (χ^2^ = 4.169, *p* = 0.244)		0 (0.0)		
Healthy	284 (55.6)	553 (53.5)	837 (54.2)	33.9
Basically healthy	201 (39.3)	425 (41.1)	626 (40.5)	32.1
Unhealthy, but can take care of themselves	25 (4.9)	44 (4.3)	69 (4.5)	36.2
Unhealthy, and cannot take care of themselves	1 (0.2)	11 (1.1)	12 (0.8)	8.3
Regional classification^***^ (χ^2^ = 81.224, *p* = 0.000)
Eastern region	325 (63.6)	828 (80.2)	1,153 (74.7)	28.2
Central region	14 (2.7)	32 (3.1)	46 (3.0)	30.4
Western region	106 (20.7)	63 (6.1)	169 (10.9)	62.7
Northeast region	66 (12.9)	110 (10.6)	176 (11.4)	37.5
Medical insurance^***^ (χ^2^ = 303.662, *p* = 0.000)
None	39 (7.6)	99 (9.6)	138 (8.9)	28.3
NCMS	272 (53.2)	539 (52.2)	811 (52.5)	33.5
URRCMI	26 (5.1)	36 (3.5)	62 (4.0)	41.9
URBMI	43 (8.4)	106 (10.3)	149 (9.7)	28.9
UEBMI	121 (23.7)	231 (22.4)	352 (22.8)	34.4
FMI	10 (2.0)	22 (2.1)	32 (2.1)	31.3
Total	511 (100.0)	1,033 (100.0)	1,544 (100)	–

* *p < 0.05;*

** *p < 0.01;*

*** *p < 0.001*.

### Migration Characteristics and Social Contacts

The median migration time was 5 years, the interquartile range (IQR) was 2–10 years, migrants with <1 year of residence accounted for 7.2% of the respondents, and 59.9% of migrants had a residence time of over 10 years. The main reasons for migrating were taking care of grandchildren (31.1%) and spending their remaining life with children (27.2%). Regarding local friends, the average number of local friends was 8.29 (SD, 11.90), but 12.9% had no local friends. With respect to exercise time, 5.2% did not exercise, and most (62.2%) of their exercise time was 60 min or less per day (Table 3). Significant differences were observed in migration characteristics and social contacts.

**Table 3:** Migration characteristics and social contacts of respondents.

	**Attending community free** ** heath checkups**		**Total (%)**	**Frequency (Yes/No) (%)**
	**Yes (%)** ** (*n* = 511)**	**No (%)** ** (*n* = 1,033)**		
Years of residence^*^ (χ^2^ = 15.163, *p* = 0.034)
1 months~	17 (3.3)	53 (5.1)	70 (4.5)	24.3
6 months~	9 (1.8)	33 (3.2)	42 (2.7)	21.4
1 year~	51 (10.0)	147 (14.2)	198 (12.8)	25.8
2 years~	57 (11.2)	103 (10.0)	160 (10.4)	35.6
3 years~	44 (8.6)	106 (10.3)	150 (9.7)	29.3
4 years~	27 (5.3)	53 (5.1)	80 (5.2)	33.8
5 years~	161 (31.5)	288 (27.9)	449 (29.1)	35.9
≥10 years	145 (28.4)	250 (24.2)	395 (25.6)	36.7
Migrating reason^**^ (χ^2^ = 19.677, *p* = 0.001)
Working or engaging in trade	90 (17.6)	207 (20.0)	297 (19.2)	69.7
Taking care of children	61 (11.9)	167 (16.2)	228 (14.8)	26.8
Taking care of grandchildren	146 (28.6)	334 (32.3)	480 (31.1)	30.4
Therapy	1 (0.2)	6 (0.6)	7 (0.5)	14.3
Spending their remaining life with children	166 (32.5)	254 (24.6)	420 (27.2)	39.5
Other reason	47 (9.2)	65 (6.3)	112 (7.3)	42
Number of local friends^***^ (χ^2^ = 59.398, *p* = 0.000)
0	23 (4.5)	176 (17.0)	199 (12.9)	11.56
1–2	74 (14.5)	174 (16.8)	248 (16.1)	29.84
3–4	76 (14.9)	157 (15.2)	233 (15.1)	32.62
5–6	95 (18.6)	167 (16.2)	262 (17.0)	36.26
7–8	37 (7.2)	54 (5.2)	91 (5.9)	40.66
9–10	90 (17.6)	146 (14.1)	236 (15.3)	38.14
Above 10	116 (22.7)	159 (15.4)	275 (17.8)	42.18
Exercise time per day^**^ (χ^2^ = 15.536, *p* = 0.008)
0	14 (2.7)	67 (6.5)	81 (5.2)	17.28
Within 30 min	116 (22.7)	236 (22.8)	352 (22.8)	32.95
31–60 min	206 (40.3)	402 (38.9)	608 (39.4)	33.88
61–90 min	29 (5.7)	33 (3.1)	62 (4.0)	47.54
91–120 min	108 (21.1)	227 (22.0)	335 (21.7)	32.24
Over 120 min	38 (7.4)	68 (6.6)	106 (6.9)	35.85
Total	511 (100)	1,033 (100)	1,544 (100)	

* *p < 0.05;*

** *p < 0.01;*

*** *p < 0.001*.

### Pathways of the Impacts of Migration Characteristics on the Utilization of Basic Public Health Services

In the model with demographic variables (model I), the result of the Hosmer and Lemeshow test was 0.778 (>0.05), which meant that the information in the current data had been completely extracted. The classification accuracy was 62.3%, suggesting that the mean regression model could correctly classify 62.3% of the observations. Age, region, and medical insurance were significant predictors of basic public health service use. Then, after adding the variables of migration characteristics (model II), the classification accuracy increased to 69.4%. We found that age was no longer significant, and years of residence and region were significant influencing factors (*p* < 0.05). Finally, social contacts were added to the model (model III), demographic variables were controlled, and the remaining variables were gradually entered into the model by forward: LR. The classification accuracy became 80.7%, which was much higher than those of model I (62.3%) and model II (69.4%). In the final model, owing to variable filtering, migration reasons were removed from the model. Furthermore, age, region, and medical insurance showed significant differences among confounders. It is worth noting that years of residence were no longer significant compared with model II, and it was replaced by the number of social contacts variables (number of local friends, exercise time per day). Comparing the results of model II and model III, we believe that, for internal elderly migrants, migration characteristics were complete mediators between social contacts and their utilization of basic public health services, which might have an effect due to the social opportunities provided for them by years of residence (Table 4).

**Table 4:** Results of binary logistic regression of the relationship between variables and the utilization of basic public health services among internal elderly migrants in China.

	**Model I**		**Model II**		**Model III**	
	**Demographic**		**Model I+** **migration characteristics**		**Model II** **+** **social contacts**	
	**OR**	**95% C. I**.	**OR**	**95% C. I**.	**OR**	**95% C. I**.
Gender	0.836	0.661–1.058	0.787	0.616–1.005	0.884	0.692–1.128
Age (years)
60–64	1.0	–	1.0		1.0	
65–69	1.275	0.959–1.649	1.138	0.860–1.504	1.363^*^	1.026–1.810
70–74	1.315	0.935–1.851	1.152	0.806–1.645	1.384	0.972–1.972
75–79	1.804^**^	1.182–2.754	1.531	0.984–2.383	2.032^**^	1.304–3.168
80–	1.782	0.940–3.380	1.449	0.734–2.859	2.136^*^	1.081–4.224
Education
Primary school or below	1.0	–	1.0		1.0	
Middle and high schools	1.258	0.982–1.611	1.190	0.904–1.570	1.209	0.919–1.590
College and above	1.363	0.935–1.988	1.164	0.757–1.791	1.205	0.786–1.847
Marital status
Married	1.0	–	1.0		1.0	
Single	0.987	0.734–1.329	1.026	0.760–1.386	1.036	0.758–1.415
Region classification
Eastern region	1.0	–	1.0		1.0	
Central region	1.328	0.690–2.557	1.538	0.788–3.001	1.487	0.742–2.980
Western region	4.650^***^	3.268–6.617	4.995^***^	3.447–7.237	4.661^***^	3.196–6.796
Northeast region	1.544^*^	1.101–2.165	1.445^*^	1.019–2.049	1.487^*^	1.044–2.119
Average monthly household income	0.846^*^	0.722–0.991	0.850	0.722–1.001	0.886	0.756–1.039
Self-reported health
Healthy	1.0	–	1.0			
Basically healthy	0.862	0.683–1.089	0.837	0.660–1.062	0.868	0.681–1.108
Unhealthy, but can take care of themselves	0.890	0.512–1.574	0.870	0.496–1.527	1.003	0.559–1.800
Cannot take care of themselves	0.104^*^	0.013–0.861	0.133	0.016–1.114	0.226	0.026–1.987
Medical insurance
None	1.0		1.0	–	1.0	–
NCMS	1.357	0.905–2.090	1.441	0.942–2.206	1.608^*^	1.043–2.478
URRCMI	2.048^*^	1.062–3.947	1.958^*^	1.022–3.855	2.338^*^	1.190–4.529
URBMI	0.979	0.569–1.684	0.988	0.570–1.714	1.084	0.620–1.859
UEBMI	1.153	0.720–1.846	1.109	0.686–1.791	1.269	0.783–2.058
FMI	0.902	0.347–2.177	0.882	0.361–2.152	0.870	0.354–2.140
Years of residence
1 month ~			1.0			
6 months~			1.001	0.383–2.618	0.958	0.356–2.580
1 year~			0.952	0.486–1.868	0.882	0.439–1.771
2 years~			1.644	0.838–3.225	1.437	0.716–2.883
3 years~			1.236	0.619–2.468	1.108	0.543–2.259
4 years~			1.653	0.774–3.532	1.454	0.665–3.179
5 years~			1.907^*^	1.027–3.544	1.669	0.878–3.170
≥10 years			1.930^*^	1.031–3.612	1.480	0.774–2.831
Migrating reason
Working or engaging in trade			1.0			
Taking care of children			1.150	0.756–1.750	–	–
Taking care of grandchildren			1.327	0.929–1.897	–	–
Therapy			0.889	0.096–8.258	–	–
Spending their remaining life with children			1.434	0.987–2.084	–	–
Other reason			1.648	0.998–2.723	–	–
Number of local friends
0					1.0	
1–2					2.988^***^	1.745–5.118
3–4					3.347^***^	1.951–5.742
5–6					3.860^***^	2.280–6.536
7–8					4.212^***^	2.209–8.031
9–10					4.222^***^	2.471–7.214
Above 10					4.350^***^	2.593–7.452
Exercise time per day
0					1.0	
Within 30 min					1.633	0.838–3.184
31–60 min					1.756	0.917–3.361
61–90 min					3.459^**^	1.511–7.919
91–120 min					1.491	0.758–2.934
Over 120 min					1.273	0.590–2.747

* *p < 0.05;*

** *p < 0.01;*

*** *p < 0.001,” –” was reference, C.I., confidence interval*.

Evidence from model III suggested a significant variation in the utilization of basic public health services across regions. Respondents in different regions had different probabilities of using basic public health services. Internal elderly migrants in the western region even had 4-fold higher odds than those in the eastern region (OR = 4.661, 95% CI: 3.196–6.796) where relatively developed cities such as Beijing, Shanghai, Guangzhou, Hangzhou, etc. are located. The reason would be that the income in the eastern region would have been greater than that in the other areas, and perhaps the individuals who earned more had less reason to access the free clinic; therefore, the utilization of paid health services in this cohort could be explored. The probability that respondents who were more than 75 years old used basic public health services was more than twice as high as that for 60–64 year-olds (OR = 2.032, 95% CI: 1.304–3.168; OR = 2.136, 95% CI: 1.081–4.224). Similarly, respondents who had social medical insurance care had a higher likelihood of utilizing basic public health services than those who did not, especially for people who had urban and rural residents cooperative medical insurance (OR = 2.338, 95% CI: 1.190–4.529).

**Figure 1:**
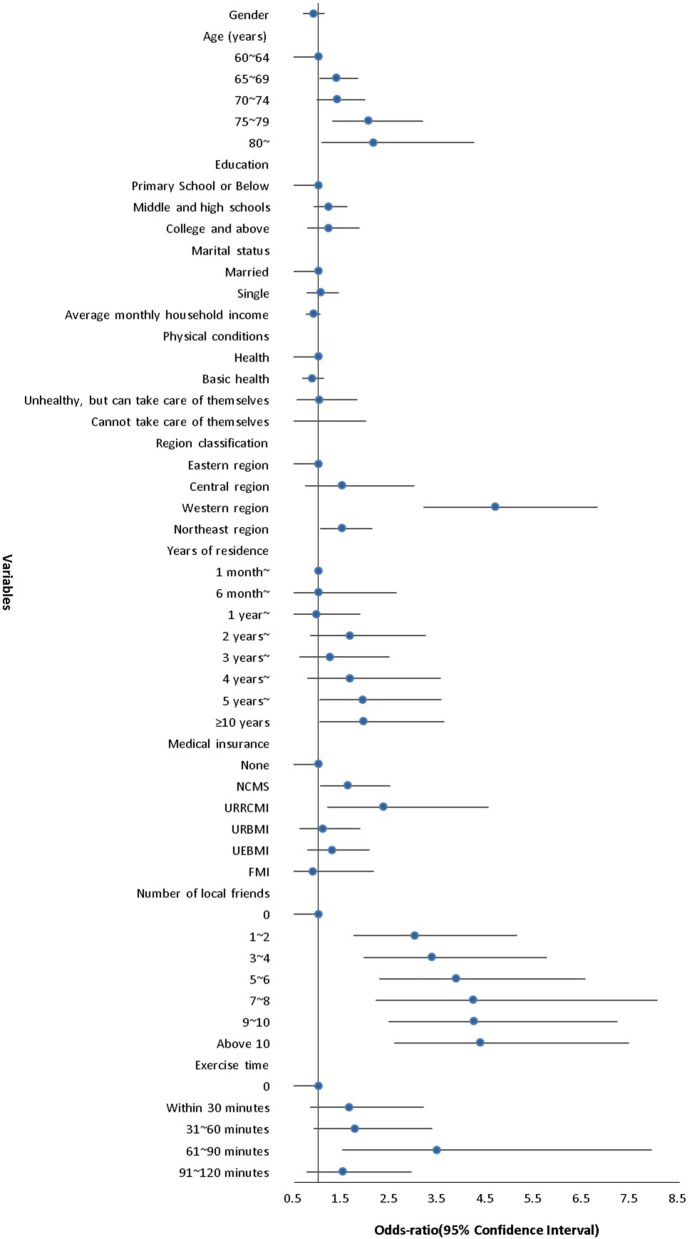
Association between variables and the utilization of basic public health services.

Regardless of the demographic variables, the associations tended to be stronger for the number of local friends than for the other factors (*p* < 0.001). Respondents who reported having local friends had almost 3-fold (OR = 2.988, 95% CI: 1.745–5.118) to more than 4-fold (OR = 4.350, 95% CI: 2.593–7.452) higher odds of utilizing basic public health services than those without local friends. In short, the more local friends a respondent possessed, the more likely the respondent was to use basic public health services. Furthermore, respondents who exercised 61–90 min per day had more than three times higher odds of utilizing basic public health services (OR = 3.459, 95% CI: 1.511–7.919) than those who did not exercise as much. In other words, internal elderly migrants who had many local friends and engaged in 61–90 min of exercise time were more inclined to use basic public health services (Table 4 and Figure 1).

## Discussion

As one would expect, when excluding the influences of demographic characteristics, migration characteristics affected the use of public health through social factors, which was similar to some other studies. In fact, we disproved the direct effect of migrating characteristics on the utilization of public health services. Social factors caused the migrating characteristics to lose their significant influence. This study supported the potential pathway of migration characteristics influencing the utilization of public health services through social factors (6–8, 11). In this study, the number of local friends and exercise time per day were significantly associated with the utilization of basic public health services rather than migration characteristics. We found that the more local friends an elderly migrant possessed, the more likely they were to use community health services, which might be due to the information and support their friends provide. Furthermore, we noted that exercise should be encouraged, as exercise time between 60 and 90 min per day was more beneficial for promoting the utilization of basic public health services.

The descriptive statistics revealed that most of the internal elderly migrants were aged 60–64. There were more men than women, and all tended to have a lower level of education, which was consistent with the characteristics of internal migrants in general (34, 35). Self-reported health was also observed. In this research, the finding regarding self-reported health was similar to previous research that most healthy people were inclined to choose to move (8). However, we did not find a correlation between health and the utilization of basic public health services, which was contradictory to other studies on migrants in general (9). This might account for the elderly individuals who were able to move away from their hometowns being more physically fit, and those who were not inclined to seek or used to seeking health services from health institutions. Another inconsistency with previous studies was the finding that marital status was irrelevant to the utilization of basic public health services. Other studies showed that persons who were 65 years of age or older and living with others were less likely to see a doctor than persons living alone (15). First, this might be due to immigration health effects; second, the companionship coming from family might have replaced the role of marriage (regarding reasons for migration, the results showed that 73.1% of the respondents in the study were accompanied by their families). Furthermore, we found that region was related to the utilization of basic public health services. This was confirmed by earlier findings of other studies that persons who lived in larger communities had a lower rate of general practitioner visits than those who lived in smaller communities (10). As mentioned above, the reason would be that the individuals with higher incomes or who were busier in metropolitan areas had less reason to access a free clinic. Additionally, a total of 8.9% of elderly internal migrants did not have medical insurance, which means they were in an inferior position compared to the 95% coverage of the entire population by three public insurance plans [NCMS, Urban Resident Basic Medical Insurance (URBMI), and Urban Employee Basic Medical Insurance (UEBMI)] (11).

As some studies showed, family migration has become a trend in the migration process in China in recent years (13), and the migration characteristics of our study supplied evidence for this trend. In addition, 12.9% of the elderly we studied had no local friends, probably because they were far from their hometowns and old friendship circles and their social circles of local friends needed to be rebuilt.

In terms of demographic characteristics, the significant predictors were age and medical insurance. We did not find that gender, marriage, education, economic income, or self-reported health had significant impacts on the utilization of basic public health services, as other studies discovered (18, 20, 36). In this case, we believe that the discrepancy may be attributed to our target research groups and the type of public health services. First, our objects were internal elderly migrants who tended to live with their families because most elderly migrants moved with their children to take care of their grandchildren; therefore, the impacts on the children might be greater than the impacts on the elderly themselves. Second, we focused on free medical check-up services in the community, so it was reasonable to suggest that, in this case, economic income was not significant.

Our data coming from secondary data were the major limitation of this study, which limited the variables that we could employ, especially in the variables of social factors. The study only contained two social contact variables. We believe that it was necessary to further explore the influence of other social factors, such as social support and social integration, which would be our next research direction. The prevalence of chronic diseases and even acute diseases were also not considered in the study. Second, this study was a cross-sectional study, so causality could not be inferred. Last, the sample size was small; furthermore, the number of internal elderly migrants in different sampling areas varied widely, which affected the representativeness of the study.

## Conclusions

To conclude, this study provided a more in-depth examination of the relationship between the studied variables and confirmed the mediating effect of social factors between migration characteristics and the utilization of basic public health services. Because only one-third of the respondents used basic public health services, some other obstacles did exist (18, 24). The findings supported the need to increase the opportunities for social contacts between local elderly individuals and internal elderly migrants (31, 37).

## Data Availability Statement

The datasets presented in this article are not readily available because the data used in this paper were provided by the National Health Commission of the People's Republic of China and we have signed a legally binding agreement with the Commission that we will not share any original data to any third parties. Requests to access the datasets should be directed to ldrkzxsj@163.com.

## Author Contributions

YL: conceived, designed, and performed the study and wrote the paper. YL, TW, and TZ: analyzed the data. All authors contributed to the article and approved the submitted version.

## Conflict of Interest

The authors declare that the research was conducted in the absence of any commercial or financial relationships that could be construed as a potential conflict of interest.

## Publisher's Note

All claims expressed in this article are solely those of the authors and do not necessarily represent those of their affiliated organizations, or those of the publisher, the editors and the reviewers. Any product that may be evaluated in this article, or claim that may be made by its manufacturer, is not guaranteed or endorsed by the publisher.

## Abbreviations

OR: odds ratios
NCMS: New Rural Cooperative Medical Scheme
URRCMI: Urban and Rural Resident Cooperative Medical Insurance
URBMI: Urban Resident Basic Medical Insurance
UEBMI: Urban Employee Basic Medical Insurance
FMI: Free Medical Insurance.
